# MicroRNA-34a: A Key Regulator in the Hallmarks of Renal Cell Carcinoma

**DOI:** 10.1155/2017/3269379

**Published:** 2017-09-20

**Authors:** Eman A. Toraih, Afaf T. Ibrahiem, Manal S. Fawzy, Mohammad H. Hussein, Saeed Awad M. Al-Qahtani, Aly A. M. Shaalan

**Affiliations:** ^1^Faculty of Medicine, Genetics Unit, Department of Histology and Cell Biology, Suez Canal University, P.O. 41522, Ismailia, Egypt; ^2^Faculty of Medicine, Department of Pathology, Mansoura University, Mansoura, Egypt; ^3^Faculty of Medicine, Department of Medical Biochemistry, Suez Canal University, P.O. 41522, Ismailia, Egypt; ^4^Faculty of Medicine, Department of Medical Biochemistry, Northern Border University, Arar, Saudi Arabia; ^5^Ministry of Health, Cairo, Egypt; ^6^Faculty of Medicine, Department of Physiology, Jazan University, Jazan, Saudi Arabia; ^7^Faculty of Medicine, Department of Histology and Cell Biology, Suez Canal University, Ismailia, Egypt; ^8^Faculty of Medicine, Department of Anatomy and Histology, Jazan University, Jazan, Saudi Arabia

## Abstract

Renal cell carcinoma (RCC) incidence has increased over the past two decades. Recent studies reported microRNAs as promising biomarkers for early cancer detection, accurate prognosis, and molecular targets for future treatment. This study aimed to evaluate the expression levels of miR-34a and 11 of its bioinformatically selected target genes and proteins to test their potential dysregulation in RCC. Quantitative real-time PCR for miR-34a and its targets; *MET* oncogene; gene-regulating apoptosis (*TP53INP2* and *DFFA*); cell proliferation (*E2F3*); and cell differentiation (*SOX2* and *TGFB3*) as well as immunohistochemical assay for VEGFA, TP53, Bcl2, TGFB1, and Ki67 protein expression have been performed in 85 FFPE RCC tumor specimens. Clinicopathological parameter correlation and in silico network analysis have also implicated. We found RCC tissues displayed significantly higher miR-34a expression level than their corresponding noncancerous tissues, particularly in chromophobic subtype. *MET* and *E2F3* were significantly upregulated, while *TP53INP2* and *SOX2* were downregulated. ROC analysis showed high diagnostic performance of miR-34a (AUC = 0.854), *MET* (AUC = 0.765), and *E2F3* (AUC = 0.761). The advanced pathological grade was associated with strong TGFB1, VEGFA, and Ki67 protein expression and absent Tp53 staining. These findings indicate miR-34a along with its putative target genes could play a role in RCC tumorigenesis and progression.

## 1. Introduction

Renal cell carcinoma (RCC) accounts for approximately 3% of human malignancies, and its incidence appears to be increasing globally [1]. RCC is not a single disease; although it is derived from cells of the renal tubular epithelium, it has several histological subtypes which differ in their clinical outcome and biological features. It is classified into clear cell RCC accounting for (75%) of cases, papillary RCC (10–15%), chromophobe RCC (5%), collecting duct RCC (<1%), and unclassified subtype [2]. For the refinement of RCC therapeutic strategies, a better realization of the RCC-underlying molecular mechanisms will be mandatory [3].

Over the past few years, emerging numerous bioinformatic tools have been developed to identify candidate disease-causing genes [4], including microRNA (miRNA) genes. This class of noncoding RNAs is small, single stranded, and 19–25 nucleotide long that act as negative regulators involved in posttranscriptional silencing of the gene expression [5]. An aberrant miRNA expression could contribute to cancer development and progression [6, 7] and could affect their target genes that are involved in many biological processes, such as cell differentiation, proliferation, apoptosis, metabolism, and development [8]. Recently, the potential therapeutic use of miRNAs has been evaluated due to their dynamic and reversible properties. This may include oncomir (oncogenic miRNA) inhibition, or tumor suppressor-miRNA replacement therapies [6, 9].

MicroRNA-34a gene (MIR-34A) that is located on chromosome 1p36 belongs to one of evolutionary-conserved miRNA families (MIR-34 family) that consists of three members: MIR-34A, MIR-34B, and MIR-34C [10]. MIR-34A has its own transcript and is expressed at higher levels than MIR-34B/C in most tissues, and this expression could be dysregulated in multiple diseases, especially in cancers [11]. It is involved in p53 pathways and is implicated in cell death/survival signaling, the cell cycle, and differentiation, thereby playing a regulatory role in carcinogenesis [12]. Previous studies have reported that several key molecules were identified as targets of miR-34a, including *Bcl-2* (B-cell lymphoma 2) [13], *TGFB* (transforming growth factor-beta) [14], the transcription inducer of cell cycle progression E2F3a [15], *MET* oncogene [16, 17], and vascular endothelial growth factor (*VEGF*) [18]. In addition, our bioinformatic analysis that has been discussed in details in Materials and Methods section of the current work has revealed other miRNA-34a-predicted target genes that could be involved in cancer-related biology, including genes for apoptosis [*TP53INP* (tumor protein p53 inducible nuclear protein), *Tp53*, and *DFFA* (DNA fragmentation factor subunit alpha)], cell proliferation (*Ki67*), and cell differentiation *SOX2* (sex-determining region Y-box 2). As miR-34a has many different targets in regulating different kinds of human cancer, Yu et al. [18] suggested the role of miR-34a is possibly tumor-specific and highly dependent on its targets in different cancer cells.

Whether miR-34a or any one of its selected aforementioned 11 putative target genes or proteins could be related to RCC pathogenesis and/or progression in our population still lacks of solid evidence. Therefore, we aimed to investigate the expression level of miR-34a and a panel of selected putative targets in an attempt to better understand the molecular mechanisms that underlie the tumorigenesis and progression of RCC. This could represent potential future therapeutic targets in renal cell carcinoma.

## 2. Materials and Methods

### 2.1. Study Population

Eighty-five archived formalin-fixed paraffin-embedded (FFPE) renal samples that have been taken from patients who underwent radical nephrectomy for a primary RCC and dating back for 3 years were collected from Pathology laboratory of Mansoura Oncology Center, Mansoura and Pathology laboratory of the Suez Canal University Hospital, Ismailia, Egypt. None of the patients received any neoadjuvant chemotherapy or radiotherapy. Complete clinicopathological data, including (patients' age, sex, and tumor's site and size), were obtained from patient medical records. Sections of cancer-free tissues adjacent to the tumor were cut, examined, and collected to serve as controls during the genetic profiling. Samples that were not homogeneous, histologically well-characterized primary renal cancer, nor had cancer-free adjacent tissues determined by an experienced pathologist have been excluded. The study was conducted in accordance with the guidelines in the Declaration of Helsinki and approved by the Medical Research Ethics Committee of Faculty of Medicine, Suez Canal University. Written informed consent was obtained from all participants before providing the archived tissue samples as part of their routine register in our University Teaching Hospitals.

### 2.2. Bioinformatic Selection of miRNA-34a and the Study Molecular Targets

**Figure 1:**
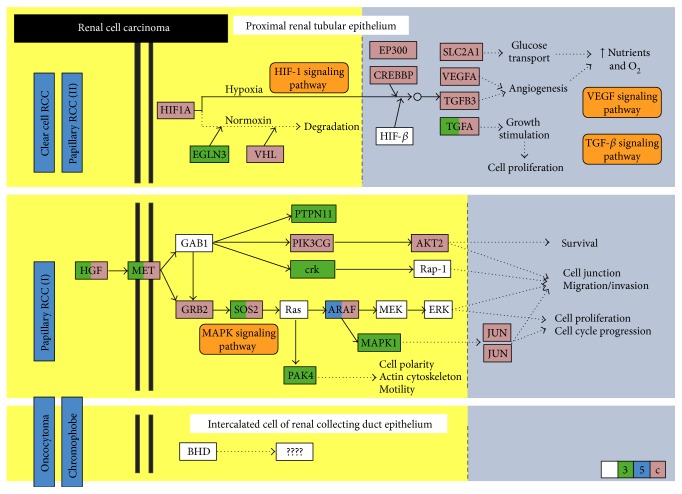
Predicted target genes of miRNA-34a in renal cell carcinoma pathway [KEGG hsa05211]. Disease pathways for each pathological subtype are shown. Hsa-miR-34a-5p can target several genes in RCC pathway. They have complementary regions at their 3′UTR, 5′UTR, or coding sequence (CDS): HIF-1, VEGF, and TGF-*β* signaling pathways in clear cell and papillary type II RCC (eosinophilic), as well as MAPK signaling pathway in papillary RCC type I (basophilic). However, candidate genes and the role of miR-34a in oncocytoma and chromophobic RCC pathways are still undetermined. Colored box: miRNA-34a target gene; green color: target on 3′UTR sequence; blue color: target on 5′UTR sequence; pink color: target on CDS; white box: not predicted target.

Predicted and experimentally validated miRNAs that significantly target renal cell carcinoma KEGG pathway (hsa05211) were identified by DIANA-mirPath v3.0 web server via Reverse Search module and TarBase v7.0 pipeline [19]. The most top and highly significant miRNA involved in this pathway was hsa-miR-34a-5p (*p* = 1.275767*e* − 88) with 28 target genes, including *MET* oncogene and three angiogenesis-related genes (*VEGFA*, *TGFB1*, *TGFB3*). Assessment of miR-34a regulatory roles in cancer biology was performed by DIANA-mirPath v3.0 online software (Figure 1).

**Figure 2:**
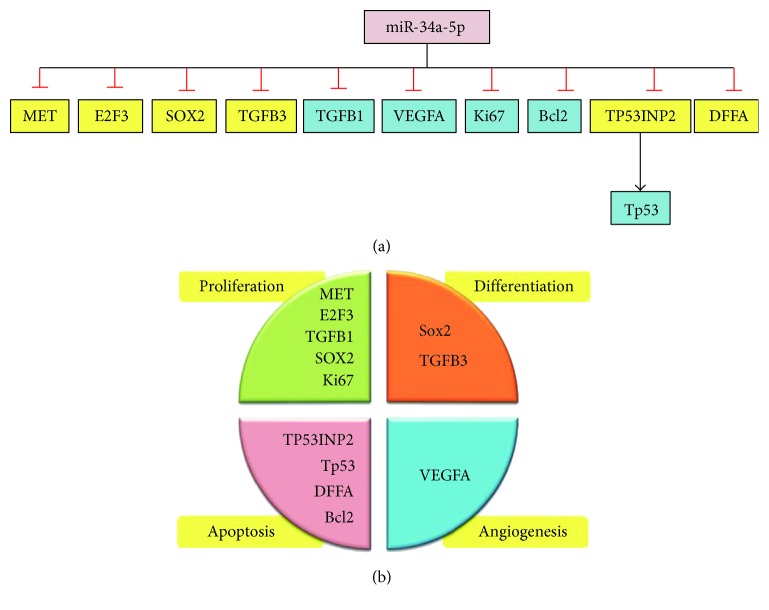
miR-34a target genes regulating the hallmarks of cancer. Eleven targets were investigated in the study. (a) List of targets analyzed by either immunohistochemistry (blue box) or quantitative real-time PCR (yellow box). (b) Classification of the miR-34a target genes and proteins according to their major role in cancer-related biology. They are enrolled in cellular differentiation, proliferation, apoptosis, and angiogenesis.

The list of all experimentally validated target genes for miR-34a-5p was retrieved from miRTarBase v20 (http://mirtarbase.mbc.nctu.edu.tw/) [20] and DIANA-TarBase v7.0 (http://diana.imis.athena-innovation.gr/) [21]. A panel of other targets involved in cancer-related biology was chosen. It included genes for apoptosis (*Tp53*, *TP53INP2*, and *DFFA*), antiapoptosis (*BCL2*), cell proliferation (*E2F3* and *Ki67*), and cell differentiation (*SOX2*) (Figure 2()). Structural analysis of MIR-34A gene and transcripts were retrieved from http://Ensembl.org. Gene expression of MIR-34A across normal human tissues was obtained from http://BioGPS.org and Expression Atlas. Complementary base pairing of miR-34a-5p seed region with the selected mRNA targets was confirmed by both http://microRNA.org resource [22] and miRTarBase v20. Prior publications demonstrating functional experimental validation of miRNA-target interactions by different methods (as luciferase reporter assay, western blot, microarray, qRT-PCR, and immunocytochemistry) are listed in Supplementary Table S1 available online at https://doi.org/10.1155/2017/3269379. A functional interaction network of selected target proteins was implemented using STRING v10 program (http://string-db.org), inferring protein-protein associations from coexpression data [23].

### 2.3. MicroRNA-34a and Gene Expression Analysis

Total RNA, including the small RNAs, was isolated from FFPE tissue sections (5 to 8 *μ*m thick) using miRNeasy FFPE Kit (Qiagen, 217504) following the protocol supplied by the manufacturer. Briefly, after the removal of paraffin by xylene and washing the sample with ethanol several times, proteins were degraded by incubation with proteinase K solution at 45°C for a few hours and later incubation with DNAses for DNA digestion. Total RNA quantity and quality were measured by Nanodrop ND-1000 (NanoDrop Technologies, Wilmington, DE). Samples with a 260/280 nm absorbance ratio less than 1.8 were discarded, and new sections of the corresponding tissue block were cut and purified, if possible. Subsequent reverse transcription (RT) and amplification of cDNA by real-time PCR using StepOne™ Real-Time PCR System (Applied Biosystems) were done as described in details previously [8, 24]. As the quantitation cycle (Cq) values of RNU6B small RNA and GAPDH were uniformly and stably expressed with no significant difference between cancer and noncancer tissues, they have been run for normalization of miRNA-34a and target gene mRNA expression analysis, respectively. All the PCR reactions were carried out in accordance with the Minimum Information for Publication of Quantitative Real-Time PCR Experiments guidelines [25]. Ten percent randomly selected study samples were reevaluated in separate runs for the study gene expressions to test the reproducibility of the qPCR which showed very close Cq value results and low standard deviations.

### 2.4. Histopathological Examination

Sections of 4 *μ*m thickness have been cut from FFPE blocks of RCC tissues for routine H&E examination, and other sections were prepared on charged slides for immunohistochemistry. Examination of three tumor slides from each specimen was done with an Olympus CX31 light microscope. Photos were obtained by a PC-driven digital camera (Olympus E-620). Cases were reviewed to determine the histological type according to the International Society of Urological Pathology (ISUP) Vancouver Modification of WHO (2004) Histologic Classification of Kidney Tumors [26]. Nuclear grade is assessed according to Fuhrman et al. [27]. Tumors were staged according to the International Union Against Cancer [28].

### 2.5. Immunohistochemistry Examination and Analysis

Immunohistochemical analysis for p53 protein, Bcl2 protein, Ki67, TGFb, and VEGF with a labelled streptavidin-biotin-peroxidase complex technique was performed on tumor sections. The primary antibodies were mouse monoclonal antibodies against p53 (clone BP-53-12, monoclonal mouse anti-human p53, c-Kit, Genemed, California, USA, diluted 1 : 50), Bcl2 (code 226M98, monoclonal mouse anti-human Bcl2, cell marque, prediluted), Ki67 (code number 1633, monoclonal mouse anti-human MIB1, DAKO corporation Carpinteria CA, USA, prediluted), TFGFB (ab9248, monoclonal mouse anti-TFGFB, abcam, USA, diluted 1 : 50), and VEGF (clone, GTX102643, monoclonal mouse anti-VEGFA, GeneTex, USA, diluted 1 : 50). A high sensitive kit has been used as a detection kit (DakoCytomation EnVision and dual link system peroxidase code K4061) using DAB as a chromogene. Antigen retrieval required pretreatment with 1 mM EDTA (at pH 8.0) for 20 minutes (p53, Bcl2, and VEGF) and 60 minutes (Ki67, and TGFb) in microwave oven. Proper positive and negative controls were performed. As a positive control, breast carcinoma has been run for p53, tonsils for Ki67, lymph node for Bcl2, and cells of proximal and distal convoluted tubules of nearby tumor-free kidney for TGFb. In addition, placental tissue was stained for VEGF as a positive control for VEGF antibody. As a negative control, sections were stained without the addition of a primary antibody.

For the immunohistochemistry assessment, examination of all prepared slides from each specimen was done with an Olympus CX31 light microscope. Photos were obtained from a PC-driven digital camera (Olympus E-620) and analyzed by Olympus Soft Imaging. Slides were scanned by ×40 magnification. Ten cellular areas were selected (i.e., the so-called hot spots) and evaluated at ×400 magnification. Positive p53 protein staining was defined as nuclear staining, and cytoplasmic staining was considered nonspecific and ignored. The percentage of tumor cell nuclei with positive staining was evaluated in relation to the total number of neoplastic nuclei in at least 10 fields observed at magnification ×400. Scoring of immunostained was categorized as mentioned in previous literature as follows: 3+ = high level (91–100% of positive cells), 2+ = medium level (11–90% of positive cells), 1+ = low level (up to 10% of positive cells), − = negative cells (0% of positive cells) [29].

Ki-67 antigen labeling was localized to the nucleus with a fine, strong, and homogenous brown granularity. Staining was considered positive if any nuclear staining was seen. Ki67 labeling index was done by calculating the ratio of positive nuclei in relation to the total number of neoplastic nuclei in 10 HPFs. Ki67 was considered to be abnormal when >10% tissue positivity was observed. The labeling index (number of positive tumor cells/total number of tumor cells expressed as a percentage) was calculated in every specimen. The Ki67 proliferation index was considered low if 0–30% of tumor cells was positive, moderate PI if 31–69% was positive, and high if ≥70% was positive. Unequivocal nuclear reactivity was considered positive [30, 31].

The BCL2 positivity was determined by cytoplasmic staining (brown) of neoplastic cells which are deep colored. The percentage of positive cells at the whole section after exclusion of the areas of reactive T cells was determined. It was scored negative if 5% or less of neoplastic cells was stained. The value of BCL2 was considered weak positive if 6% to less than 50% was brown stained, and strong positive if ≥50% of tumor cells was brown stained [32].

TGFB immunohistochemistry specimens were classified based on the intensity of staining as follows: weak or absent staining (< 10% of cells), intermediate (10–25%), focally strong (25–50%), and strong (> 50% of cells) [33]. VEGF sections were considered positive for VEGF if the membranes or cytoplasm of more than 10% of tumor cells was stained [34].

### 2.6. Statistical Analysis

Data were managed using the R package (version 3.3.2). Categorical variables were compared using the chi-square (χ^2^) or Fisher's exact tests where appropriate, while Mann–Whitney *U* (MW) and Kruskal-Wallis (KW) tests were used to compare continuous variables. The correlation between miR-34a level and mRNAs and protein expression was calculated by Spearman's rank correlation analysis. A two-tailed *p* value of < 0.05 was considered statistically significant. The receiver operating characteristic (ROC) curves were performed to get the best cutoff values of either miR-34a or mRNAs for discriminating RCC from noncancer tissues. The fold change of miRNA and mRNA expressions in each patient cancer tissue relative to the corresponding cancer-free tissue was calculated via Livak method based on the quantitative cycle (Cq) values with the following equation: relative quantity = 2^−ΔΔCq^, where ΔΔC_q_ = (C_q_ miRNA–C_q_ NBU6)_RCC_ − (C_q_ miRNA–C_q_ NBU6)_NAT_ in case of miR-34a analysis and where ΔΔC_q_ = (C_q_ mRNA–C_q_ GAPDH)_RCC_ − (C_q_ mRNA–C_q_ GAPDH)_NAT_ in case of study gene target analysis [35].

## 3. Results

### 3.1. Baseline Characteristics of the Study Population

In the current study, 85 patients (32 females and 53 males) were enrolled in the study. Their age ranged from 20 to 79 years old with mean ± SD of 52.23 ± 11.12. Renal cancer samples were compared to normal tissues. There was no significant difference in age and gender between FFPE tumor samples and normal renal tissues (*p* = 0.087 and *p* = 0.214, resp.). The clinicopathological characteristics of renal cell carcinoma patients are demonstrated in Table 1. According to the 2004 WHO classification, several histological RCC subtypes were recognized in the study population. The most frequent histological subtypes included clear cell renal cell carcinomas (ccRCC), papillary renal cell carcinomas (pRCC), and chromophobe renal cell carcinomas (crRCC). Most cancer specimens were moderately or poorly differentiated; nevertheless, low proportions of tumors had high tumor size (T3), positive lymph node involvement, capsular and pelvic infiltration, and vascular invasion.

### 3.2. Gene and Protein Expression Analysis

**Figure 3:**
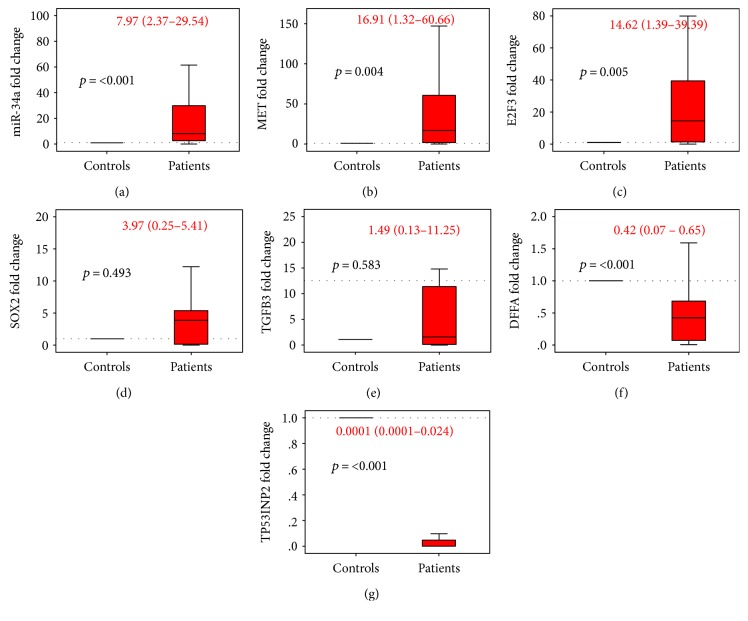
Gene expression profiling in cancer and normal renal tissues. Data are represented as medians. The box defines upper and lower quartiles (25% and 75%, resp.), and the error bars indicate upper and lower adjacent limits. Expression levels of miR-34a and targets in cancer and normal tissues were normalized to RNU6B and GAPDH, respectively. Fold change was calculated using the delta-delta CT method (2^−ΔΔCT^) in comparison to normal renal tissues. The gray dash line represents the expression level of normal renal tissues (equivalent to 1.0). *p* values < 0.05 were considered statistical significant. Mann–Whitney *U* test was used for comparison. Median and quartile values of patients are noted in red.

Using qRT-PCR technology and immunohistochemistry, gene and protein expression analyses were used to identify differential molecular changes between tumor and normal renal tissues. Gene expression profiling revealed a significant overexpression of miR-34a in almost all RCC patients (91.7%) with an overall median and quartile values of 7.97 (2.37–29.54). In addition, among the 6 genes that have been predicted to be targeted by miR-34a via the in silico computational tools, two genes were significantly upregulated (*MET* and *E2F3*) in 87.1% of FFPE samples, while two others were downregulated (*TP53INP2* and *SOX2*) in almost all RCC patients compared to noncancer tissues (Figure 3). However, correlation analysis revealed no significant relationship of miR-34a with the tested target genes (Supplementary Table S2 and Figure S1).

**Figure 4:**
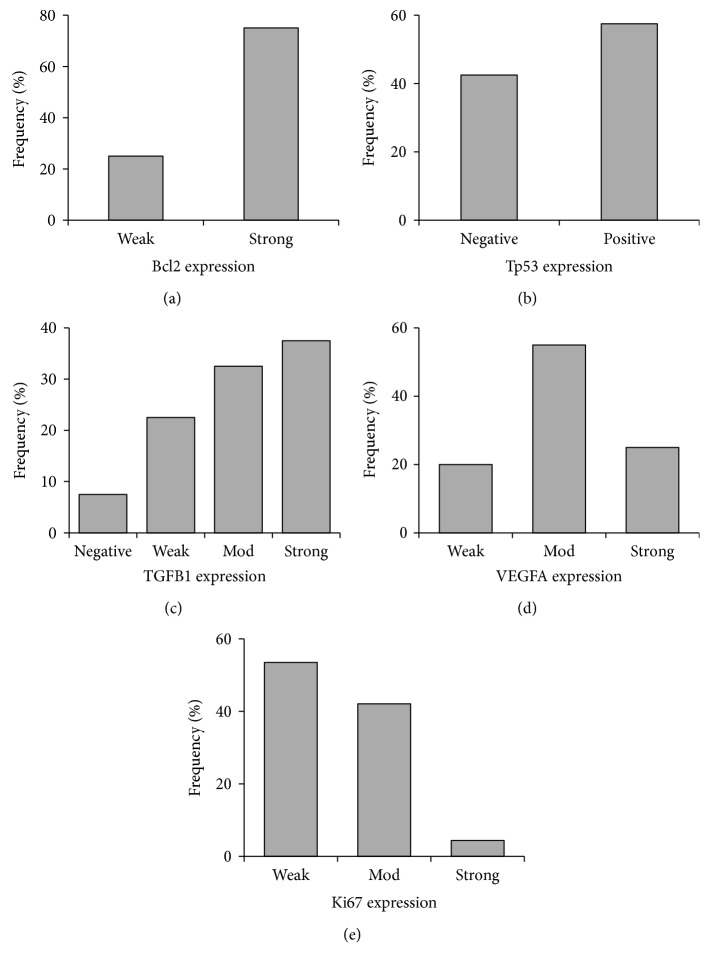
Frequency of Immunohistochemistry markers of miR-34a putative target proteins in RCC specimens. Five protein markers were examined, Bcl2, Tp53, TGFB1, VEGFA, and Ki67.

Immunohistochemistry of renal tissue samples demonstrated variable staining patterns (Figure 4()). Ki-67 expression, a cell proliferation marker, was detected in all cases of RCC but with variable level of expression. Low level of expression (<10%) was detected in 28 cases (70%), while high expression (≥10%) was noted in 12 cases (30%). Similarly, the angiogenesis-mediated protein (VEGFA) and the antiapoptotic marker (Bcl2) were expressed in all cancer tissues. Eighty percent of patients had high expression of VEGFA, while only 20% demonstrated weak staining. Antihuman Bcl2 antibody was widely distributed all over the renal cancer tissues. Strong expression was noted in 75% of samples. For TGFB1 protein, most of cancer tissue attained moderate to strong expression in the cytoplasm, but the protein was less intense in approximately one-fifth of patients and absent in three samples only. In contrast, expression of the tumor suppressor protein (Tp53) was not detected by immunohistochemistry in less than half of tumor specimens. Unlike tumor cells, which had nuclear staining, lining cells of the proximal tubules stained the cytoplasm only.

ROC curve analysis of all genes and proteins showed significant high diagnostic performance of miR-34a (AUC = 0.854), *MET* (AUC = 0.765), and *E2F3* (AUC = 0.761) in differentiating between cancer specimens and noncancer tissues (Table 2).

### 3.3. Association of Gene and Protein Signature with Clinicopathological Features

**Figure 5:**
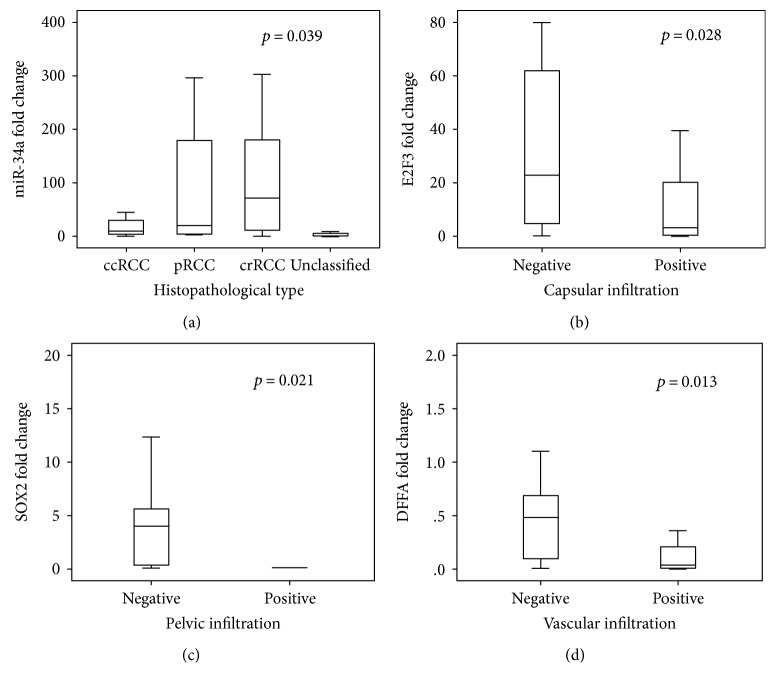
Association of miR-34a and target genes with the clinicopathological features in RCC patients. (a) Higher expression of miR-34a was significantly associated with chromophobic RCC subtype. (b) Lower levels of *E2F3* were associated with capsular infiltration. (c) Lower levels of *SOX2* had higher frequency of pelvic infiltration in RCC tumor tissues. (d) *DFFA* downregulation was associated with vascular infiltration.

The expression of miR-34a was markedly higher in RCC samples with chromophobic renal cell carcinoma and lower in clear cell type (*p* = 0.039) (Figure 5(a)). In addition, its level was inversely correlated with the tumor pathological grade (*r* = −0.301, *p* = 0.037). Among the target genes, lower levels of three genes *E2F3*, *SOX2*, and *DFFA* were significantly associated with capsular, pelvic, and vascular invasion, respectively (Figures 5(b), 5(c), and 5(d)). These findings were consistent with Spearman's correlation analysis (Supplementary Table S3).

**Figure 6:**
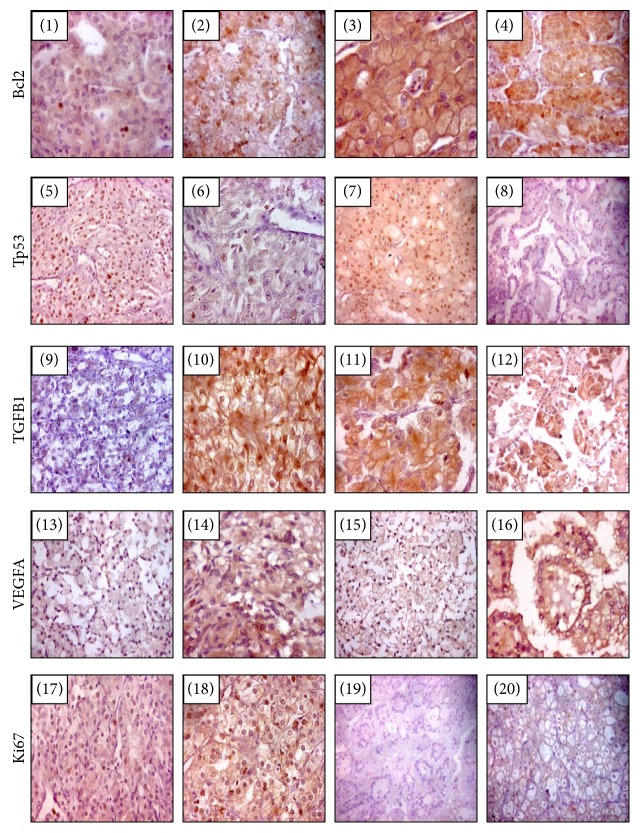
Immunohistochemistry images according to type and grade of RCC. Clear cell RCC (Ng1) weakly expresses bcl2 (x100), (photo 1), clear cell RCC (Ng2) with focal strong cytoplasmic bcl2 (x100) (photo 2), chromophobe RCC (Ng2) with diffuse moderate expression of bcl2 (x400) (photo 3), and papillary RCC (Ng2) with diffuse strong expression of bcl2 (x200) (photo 4). Clear cell RCC (Ng1/2) with diffuse strong nuclear expression of P53 (x100) (photo 5), clear cell RCC clear cell RCC (Ng3) showed scarce cell nuclei express P53 (X200) (photo 6), chromophobe RCC (Ng2) diffusely and strongly express P53 (X100) (photo 7), and papillary RCC do not express p53 (x100) (photo 8). Clear cell RCC (Ng2) with weak expression of TGFB1 (x100) (photo 9), RCC (Ng3) with strong expression of TGFB1 (x200) (photo 10), chromophobe RCC with focal strong expression of TGFB1 (X200) (photo 11), and papillary with focal strong expression of TGFB1 (X200) (photo 12). Clear cell RCC (Ng1) with diffuse weak expression of VEGFR (X100) (photo 13), clear RCC (Ng3) with diffuse moderate expression of VEGFR (X200) (photo 14), chromophobe RCC with weak expression of VEGF (X100) (photo 15), papillary RCC with intermediate expression of VEGFR (X200) (photo 16). RCC (Ng2) with low expression of Ki67 (X100) (photo 17), RCC (Ng3) with high expression of Ki67 (X200) (photo 18), and papillary RCC does not express Ki 67 (x100) (photo 19), and chromophobe RCC do not express Ki67 (x200) (photo 20).

**Figure 7:**
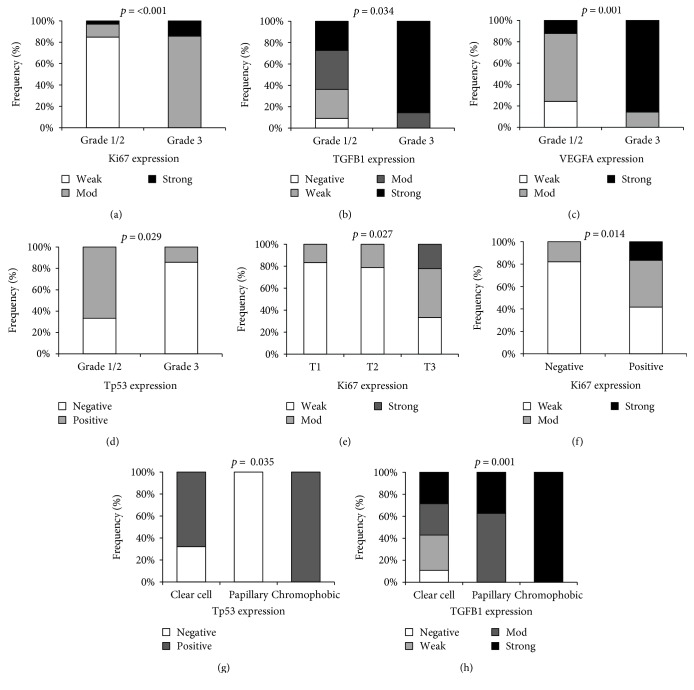
Association of immunohistochemistry markers with the clinicopathological features in RCC patients. The figures illustrated higher intensity of Ki67, TGFB1, VEGFA, and Tp53 in RCC tumors with advanced pathological grade (a–d), extensive staining of Ki67 antibodies in T3 samples and capsular infiltration (e-f), and differential expression of Tp53 and TGFB1 in various histopathological subtypes (g-h).

Immunohistochemistry photos of the target proteins in renal tissues in relation with pathological parameters are illustrated in Figure 6. The advanced pathological grade was significantly associated with strong expression of Ki67 (*p* = 0.001), TGFB1 (*p* = 0.034), VEGFA proteins (*p* = 0.001), and absent Tp53 staining (*p* = 0.029) (Figures 7(a), 7(b), 7(c), and 7(d)). Larger tumor size and capsular infiltration also showed higher Ki67 expression (*p* = 0.027 and p = 0.014, resp.) (Figures 7(e) and 7(f)). Additionally, there was differential expression of TGFB1 and Tp53 proteins according to the specimen histopathological diagnosis, with stronger staining in chromophobic renal cell carcinoma type (Figures 7(g) and 7(h)). Similarly, correlation analysis between proteins and clinicopathological characteristics demonstrated moderate correlation of Ki67 with histopathological diagnosis (*r* = −0.419, *p* = 0.007), grade (*r* = 0.690, *p* < 0.001), tumor size (*r* = 0.389, *p* ≤ 0.001), LN invasion (*r* = 0.351, *p* = 0.026), and capsular infiltration (*r* = 0.431, *p* = 0.006). TGFB1 protein showed moderate correlation with histopathological diagnosis (*r* = 0.427, *p* = 0.006) and tumor grade (*r* = 0.441, *p* = 0.004). VEGFA protein also showed a significant positive correlation with pathological grade (*r* = 0.563, *p* < 0.001). In contrast, there was a negative correlation between Tp53 and grade (*r* = −0.403, *p* = 0.010) (Supplementary Table S3).

### 3.4. In Silico Data Analysis

**Figure 8:**
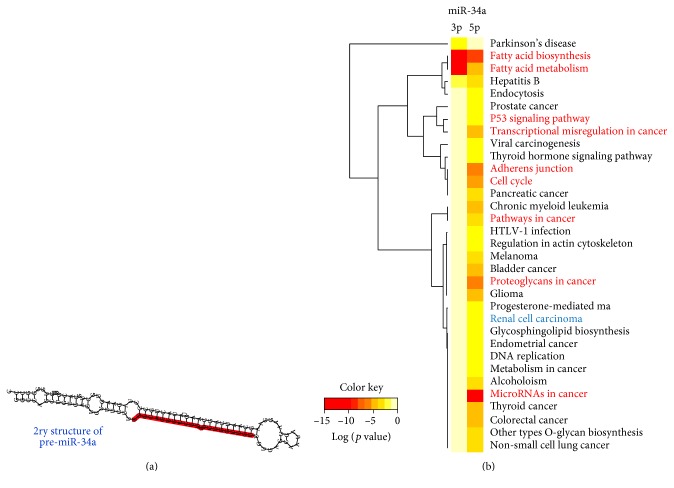
Structural analysis of MIR-34A gene locus and transcripts. (a) *Homo sapiens* hairpin secondary structure of pre-miR-34a stem-loop. Mature miR-34a-5p highlighted in red [data source: miRTarBase v20]. (b) Enrichment pathway analysis for miR-34a. Heat map showing targeted pathways, diseases, and cancers for both hsa-miR-34a-5p and hsa-miR-34a-3p with *p* values < 0.05 and microT-CDS threshold 0.4. A total of 33 pathways have target genes (in CDS or 3′ UTR regions) for miR-34a. The enrichment results of validated target genes showed that most pathways were related to cancer and cancer-related pathways. KEGG pathway annotations related specifically to current RCC work had been labeled (red). Degree of color is based on the significant *p* values of the predicted algorithm by DIANA tools; the red has the top significance estimated by false discovery rate (data source: DIANA-miRPath v2.0 web server).

Hsa-miR-34a is encoded by MIR-34A gene (ENSG00000284357), mapped at 1p36.22. The gene has a single exon which contains a p53-binding site within a CpG island about 30 kb upstream of the mature MIR-34A sequence and encodes for a transcript of 110 bp in length. The precursor miRNA stem-loop is processed in the cytoplasm of the cell, with the predominant miR-34a mature sequence excised from the 5′ arm of the hairpin. Secondary structure of hsa-miR-34a stem-loop predicted by computational programs is illustrated in Figure 8(a). Functional characterization of miR-34a based on differential expression experiments revealed its control on numerous cancer-related molecular pathways and cellular processes. It can control up to 115 genes involved in pathways in cancer (hsa05200), in addition to dozens of genes in particular tumor types (Figure 8(b)). As shown in the heat map, the most top five significant pathways targeted by miR-34a were microRNAs in cancer, fatty acid biosynthesis, proteoglycans in cancer, adherence junction, and cell cycle (Figure 8(b) and Supplementary Table S4).

Interaction of mature miR-34a-5p with complementary sites of selected experimentally validated targets is shown in Supplementary Figure S2. Protein-protein interaction between the targets is shown in Supplementary Figure S3. Enrichment analysis of the target panel elucidated their functional impact on numerous biological processes and cancer KEGG pathways (Supplementary Table S5 and S6).

## 4. Discussion

A key goal in clinical oncology is the development of therapeutic strategies that impede specific deregulated biological pathways in cancer. Understanding these pathways which involve candidate disease-causing genes will provide new therapeutic modalities for renal cancer.

In the current study, upregulation of miR-34a was observed in more than 90% of RCC patients, with median fold change of 7.97 in RCC FFPE tissues compared to noncancer tissues. ROC analysis revealed a high diagnostic performance of miR-34a in discriminating between cancer and noncancer tissues. However, higher levels showed a better prognosis (i.e., it was moderately correlated with well differentiated tumors). In addition, expression profiles in chromophobic RCC samples were markedly greater than that of clear cell and papillary subtypes.

According to a survey across diverse normal human tissues, miR-34a was downregulated in most human normal tissues, including renal cortex and medulla (data source: U133plus2 Affymetrix microarray from http://BioGPS.org). Consistent with our findings in renal cancer tissues, miRNA-34a has been reported to support cell proliferation in oxidative stress-induced renal carcinogenesis rat model [36] and it has been found to be overexpressed in various types of human cancer [37–40]. As one of the upregulated miRNAs in RCC, it has been speculated to function by downregulating tumor suppressor genes including secreted frizzled-related protein 1 (SFRP1) [41]. In addition, miR-34a was identified to be a direct target of the tumor suppressor Tp53 protein in human and mouse cells and mediates some of its proapoptotic biological functions [42, 43]. Similarly, He et al. found that deregulation of miR-34a on response to DNA damage and oncogenic stress depends on p53 *in vitro* and *in vivo* [13].

*In vitro*, miR-34a was coexpressed with Tp53 at high levels in colorectal cancer cell lines and in irradiated mice but was not expressed in Tp53-knockout mice [44]. These findings could explain the good prognosis that is implied by higher levels of miR-34a in the current samples with low pathological grade and in chromophobic RCC subtype. It has been found that miR-34a expression could suppress the cell proliferation [38, 44], promote apoptosis through the induction of caspase-dependent apoptotic pathways [42, 45] in several cancer cell lines [46–48], and cause dramatic reprogramming of gene targets that regulate apoptosis, DNA repair, cell cycle progression, epithelial-mesenchymal transition, and angiogenesis [42, 49]. In addition, miR-34a restoration in cancer cells was shown to induce cell cycle arrest at G1 and G2/M phases and sensitized the cells to chemo- and radiotherapy [16, 50]. However, low expression of miR-34a was noted in other types of cancer [46, 51–54], in glioblastoma and glioma with mutant Tp53 [55], in chronic lymphocytic lymphoma with Tp53 deletion [56], and in metastatic hepatocellular carcinoma [57], reflecting that miRNA-34a can work in a cell type-specific manner with a differential p53 pathway inactivation [36].

Taken our results with the findings of prior studies, we could support the hypothesis that miR-34a overexpression in the current study is a secondary consequence in cancer cells elucidated to compete the DNA damage and uncontrolled growth proliferation. Accumulation of further mutations in higher pathological grade tumors, especially those related to Tp53 gene activity or 1p36 locus itself, could account for the fall of miR-34a expression profile in those patients. Further functional studies are recommended to unravel the molecular mechanisms underlying the chromophobic RCC which has the best prognosis among all other subtypes in our cases [58–60].

In silico analysis of miR-34a targets in databases revealed numerous candidate gene targets. Functional annotation and enrichment analysis showed high linkage of miR-34a with cancer-related pathways. It can influence several pathways involved in all cancer hallmarks acquired during the multistep development of human tumors, by sustaining proliferative cell signaling, evading growth suppressors, resisting apoptosis, inducing angiogenesis, and activating invasion and metastasis. In the current study, we identified predicted putative miR-34a binding sites within the 3′ UTR, 5′UTR, or coding regions of eleven mRNAs. These selected genes were functionally validated in prior experiments listed in Supplementary Table S1. Nevertheless, our data did not reveal inverse correlations of these targets with miR-34a. This could be explained by the fact that their gene expressions result from several integrated cell responses and cross talk between signaling pathways. Additionally, according to miRNA databases, there are multiple-to-multiple relationships between microRNAs and target genes; one miRNA may regulate transcription of many genes, and a single gene could be targeted by multiple miRNAs simultaneously, thus forming complex genetic circuits in human cancer [61, 62]. Liu et al., in addition, speculated the identified putative targets of miRNA could be regulated by translation inhibition rather than degradation. Subsequently, this would leave mRNA levels unaffected but reduce the protein levels. This speculation warrants the need of protein level measurement in tumor/normal samples along with the miRNA and mRNA levels for the same gene to identify such type of regulation [41].

Of the deregulated target genes expressed significantly in the current renal cancer specimens, *MET* and *E2F3* were significantly upregulated in RCC compared to noncancer tissues with high diagnostic performance. The *Met* protooncogene, mapped at 7q31.2, has two alternative spliced isoforms (http://genecards.org). We identified two putative miR-34a binding sites within the 3′ UTR and 5′ UTR of the human c-Met mRNA. Similarly, Li et al. [16] and Hu et al. [17] reported that c-Met is directly targeted by miR-34a. The *MET* gene encodes a receptor tyrosine kinase that is activated by hepatocyte growth factor (HGF) [16]. Ligand binding at the cell surface induces autophosphorylation of carboxyl terminus of MET on its intracellular domain that generates docking sites for second messengers, which activate several signaling pathways involving RAS-ERK, mitogen-activated protein kinase (MAPK), phosphatidylinositol 3-kinase- (PI3K-) AKT, signal transducer and activator of transcription (STAT), and phospholipase C [63]. Such downstream signaling pathways evoke a variety of pleiotropic physiological processes, including survival, morphogenesis, differentiation, epithelial-mesenchymal transition, and regulation of cell migration [64]. However, improper activation of c-MET may confer proliferative and invasive/metastatic abilities of cancer cells [65]. Similar to our findings in RCC patients, MET overexpression was associated with multiple human cancers [63, 66–71]. Its aberrant expression by different mechanisms, including point mutations [72], gene amplification [73], and oncogenic deletion [74, 75, 76], may lead to a more aggressive cancer phenotype and may be a prognostic indicator of poor overall survival and resistance to therapy [74–76].

The second upregulated gene in most of the current RCC samples was the transcription factor *E2F3*. ROC analysis showed its high discrimination accuracy in RCC diagnosis. In addition, its expression profile was associated with capsular infiltration. *E2F3* gene lies on chromosome 6p22.3 and has 3 transcript variants encoding 3 proteins of 465, 334, and 128 amino acids long. In contrast to full-length E2F3 protein, which is expressed only at the G1/S boundary, truncated isoforms are expressed throughout the cell cycle [77, 78]. E2F3 recognizes a specific sequence motif in DNA and interacts directly with the tumor suppressor retinoblastoma protein (pRB) to regulate the expression of genes involved in the G1/S boundary of the cell cycle and DNA replication; hence, E2F3 has a critical role in the control of cellular proliferation [79]. Acute loss of E2F3 activity affected the expression of genes encoding DNA replication and mitotic activities [80]. *In vitro* and *in vivo* studies showed a failure of division and proliferation in E2F3-null retinal progenitor cells [81] and early embryonic death in E2F3-null mice [82, 83]. Dysregulation of *E2F3* and altered copy number and activity of this gene have been observed in a number of malignant tumors [84–92] and correlated with several pathological features of cancer like the pathological grade and tumor cell proliferation rate [91], as well as tumor aggressiveness and poor overall survival [91, 92]. However, transgenic mice expressing inducible *E2F3* resulted in hyperplasia, but not tumor development [93], supporting its role in tumor progression rather than initiation.

As an excellent marker to determine the growth fraction of a given cell population, the expression of proliferation-related Ki67 antigen was investigated in the current study. The fraction of Ki67 positive cells is often correlated with the clinical course of the tumors. Currently, this marker of proliferation was detected in all cases of RCC, but with variable levels of expression. High expression of Ki67 was associated with advanced pathological grade, large tumor size, lymphatic invasion, and capsular infiltration. In addition, strong staining was correlated with chromophobic RCC subtype. Antigen Ki67 is a nuclear protein, encoded by *MKI67* gene that is mapped at 10q26.2. It has five splice variants; only two of them are translated to synthesize a long form (3256 aa/ 395 kDa) and a short form (2896 aa/345 kDa) proteins that differ only by the presence or absence of exon 7 [94]. Antigen Ki67 is associated with cellular proliferation [95]. During interphase, the Ki67 protein can be exclusively detected within the cell nucleus, whereas in mitosis, most of the protein is relocated to the surface of the chromosomes [96]. Ki67 protein is present during all active phases of the cell cycle (G1, S, G2, and mitosis) but is absent from resting cells (G0) [95, 97]. GO annotation identified the gene to play vital roles in chromosomal segregation regulation and organization, nuclear division, cell cycle, organ regeneration, and stress response (http://genecards.org). It is also associated with ribosomal RNA transcription and synthesis [98, 99]. Several lines of evidence have implicated the importance of Ki67 index in multiple cancers [100–106] and reported its prognostic value for survival and tumor recurrence [107–114].

Regarding the TGFB superfamily proteins, they were known to be implicated in cell growth and differentiation. These growth factors bind various TGF-beta receptors, leading to recruitment and activation of SMAD family transcription factors which regulate the expression of the downstream genes, including interferon gamma and tumor necrosis factor alpha [115]. We examined the expression profile of TGFB1 protein and *TGFB3* gene. In RCC samples, *TGFB3* mRNA did not show differential expression compared to noncancer tissues. However, TGFB1 protein showed moderate to strong cytoplasmic staining in most samples. Higher protein level expression was associated with poor tumor differentiation and chromophobic subtype. This protein is involved in embryogenesis and cell differentiation and may play an important role in apoptosis, immune defense, inflammation, and tissue repair [115, 116]. Overexpression or alterations of its active protein induced by gene somatic mutations were frequently observed in several tumor cells [117–121] and were correlated with tumor aggressiveness, invasion, angiogenesis, metastasis, immune surveillance inhibition [122], and epithelial-mesenchymal transformation [117].

SOX2 is a critical transcription factor for self-renewal and maintenance of undifferentiated embryonic stem cells [123]. *SOX2* gene is mapped at 3q26.33, consisting of a single exon that encodes a protein of 318 amino acid residues (http://genecards.org). It was reported to be involved in embryonic development regulation and in the cell fate determination, and its over expression can induce reprogramming of somatic cells to acquire pluripotency characteristics [124, 125]. SOX2 was identified as an oncogenic factor and was reported to be overexpressed in certain types of cancer [123, 126–129]. Knockdown of *SOX2* could inhibit cell viability and tumorigenesis *in vitro* and *in vivo* [123, 126] by potentiating cell cycle arrest associated with decreased levels of CCND1 and phosphorylated Rb and/or by upregulating of p27Kip1 level [130]. In our samples, no differential expression was observed between cancer and noncancer tissues; nevertheless, lower expression of *SOX2* mRNA was correlated with cancer infiltration of renal pelvic tissues.

In the current study, one antiapoptotic and three proapoptotic miR-34a targets regulating essential cancer-related pathways were examined. The apoptotic regulator *BCL2* gene, mapped at 18q21.33, has 2 alternative transcripts and encodes an integral outer mitochondrial membrane protein [131]. It has two protein isoforms, Bcl2a (5.5-Kb mRNA/239 aa) and Bcl2b (3.5-Kb/205 aa), which are identical except for the C-terminal portion. The former contains a hydrophobic tail for membrane anchorage which seems to be necessary for antiapoptotic ability [132]. Bcl2 is found on the outer membrane of mitochondria. It functions as an apoptosis inhibitor by forming complexes with caspase-9 and APAF1, thus prevent them to initiate the protease cascade and apoptosis through caspase-3 cytochrome C-dependent activation [133]. In 2002, Marsden et al. [134] discovered that Bcl2 can also function independently via other pathways. Bcl2 constitutively blocks p53-induced apoptosis and enables the survival of colorectal cancer cells [135]. Overexpression of Bcl2 blocks TNF-related apoptosis-inducing ligand- (TRAIL-) induced apoptosis in human lung cancer cells [136]. In addition to the antiapoptotic function, Bcl2 is known to regulate mitochondrial fusion and fission dynamics [137]. Bcl2 acts as a potent regulator of cell survival in neurons both during development and throughout adult life [138]. In cancer, overexpression of the antiapoptotic Bcl2 can result in a distinct cellular growth advantage due to lack of cell death, a hallmark of cancer. In the present study, Bcl2 protein was expressed in all cancer tissues. Similarly, in previous studies, Bcl2 upregulation has been reported in many types of cancer [139–142]. Moreover, it has also been associated with poor clinical outcome and shorter overall survival in cancer patients [143]. It can confer resistance to chemotherapy and radiotherapy in some types of cancer [144, 145]. In addition, targeting Bcl2 by miR-125a, miR-206, and miR-34a was reported to inhibit the cell proliferation and induce apoptosis in multiple cancer cells [146–148].

The tumor suppressor protein Tp53, the guardian of the genome, is essential for the carcinogenesis prevention. In the current study, it was not detected by immunohistochemistry in less than one half of the specimens. Absent staining of Tp53 protein in tumor cell nuclei was significantly associated with advanced pathological grade, while positive staining was observed in the chromophobic RCC, known to have the best prognosis. The transcription factor Tp53 is encoded by *TP53* gene mapped at 17p13.1. This gene has a complex transcriptional expression pattern encoding 28 different mRNA variants through the use of an internal promoter in intron 4 and alternative splicing machinery. All variants could be detected in all tissues, and only 5 is exclusively transcribed in tissue-specific manner [149]; each isoform has distinct biological activity and subcellular localizations [150]. Normally, Tp53 is expressed at low levels and kept inactive through the action of MDM2 (mouse double minute 2 homolog) which promotes its degradation [150]. However, during cellular stresses or DNA damage, activated Tp53 induces cell cycle arrest for DNA repair or force apoptosis. It binds to DNA and regulates transcription of target genes that induce cell cycle arrest, apoptosis, and DNA repair [150–152]. It can trigger cell death independently of its transcriptional activity through subcellular translocation and activation of proapoptotic Bcl-2 family members [153]. Attenuation of Tp53 activity would render the cells more susceptible to further genetic damage and therefore to neoplastic transformation and tumor progression.

Another apoptotic gene, *Tp53INP2*, is located at 20q11.22 with 4 transcripts and encodes for 3 putative protein variants of 220, 88, and 77 amino acid long. It is thought to be a scaffold protein that is normally expressed upon induction by the Tp53 protein [154]. The protein encoded by this gene has two distinct functions depending on its cellular localization [155]. It is essential for proper autophagy, a self-degradative process that occurs at critical times in development to recycle unnecessary intracellular components and damaged organelles [156]. Tp53INP2 protein shuttles between the nucleus and the cytoplasm, depending on cellular stress conditions, and relocates in the autophagosomes during autophagy activation. It recruits Atg8-like proteins to the autophagosome membrane by interacting with the transmembrane protein VMP1 (vacuole membrane protein 1) [154]. Failure of autophagy is thought to be one of the main reasons for the accumulation of cell damage and aging [157]. In addition to its role in autophagy, it serves as a transcriptional coactivator for several nuclear receptors, such as the glucocorticoid receptor, vitamin D receptor (VDR), and peroxisome proliferator-activated receptor gamma [155], thus possess a tumor suppressor-like functionality similar to Tp53 [158, 159]. Dysregulation of *Tp53INP2* expression was found differently in several types of cancer tissues [160–164]. Therefore, our results along with previous data highlight its putative role in cancer development and progression.

Low levels of the apoptotic gene, *DFFA*, were observed in almost all RCC samples. Lower expression was associated with vascular infiltration. *DFFA* gene is located in the same region of miR-34a at 1p36.22 which is commonly deleted in human tumors. DFFA plays an essential role in apoptosis. When cleaved by caspase-3, it induces the release of its partner DFFB, which in turn triggers DNA fragmentation by its nuclease activity [165]. Hence, absence of this protein could result in aberrant apoptosis, invasive growth, and metastasis [166]. Similar to our findings, downregulated DFFA expression was observed during the exponential phase of growth in several human colonic cancer cell lines [167]. DFFA (−/−) mice exerted severe genomic instability and tumor progression in colon epithelial cells [168]. Moreover, low DFFA expression was associated with poor prognosis in esophageal cancer [169] and neuroblastoma tumors [170].

As angiogenesis is of central importance in the growth and metastasis of tumors [171], we investigated the expression of the angiogenesis-mediated protein, VEGFA in RCC compared to noncancer tissues. VEGFA protein expression was detected in all RCC tissues, with 80% of samples showing strong staining. Elevated levels were associated with advanced tumor grade. VEGFA protein is encoded by the *VEGFA* gene, mapped at 6p21.1, a highly polymorphic region that showed association with cancer susceptibility, aggressiveness, and therapeutic response in various tumor types [172, 173]. Alternative exon splicing can generate up to 29 transcript variants with different isoforms (http://Ensembl.org). There was also evidence for alternative translation initiation codons resulting in additional isoforms. VEGFA promotes proliferation and migration of vascular endothelial cells both *in vitro* and *in vivo* and is essential for both physiological and pathological angiogenesis [172]. This prosurvival effect is mediated via PI3-kinase/Akt signal transduction pathway [174]. In addition, it induces permeabilization of blood vessels, thus known as a vascular permeability factor [175]. It induces endothelial fenestration in vascular beds [176] and enhances vasodilatation *in vitro* in a dose-dependent manner [177]. In addition, VEGFA promotes apoptosis and induces expression of the antiapoptotic protein Bcl-2 [178]. *In vivo*, VEGFA inhibition results in abnormal embryonic blood vessel formation and extensive apoptotic changes in the vasculature of neonatal mice [179, 180]. Within tumors, cancer cells and cancer-associated stroma are the major source of VEGFA [173]. It influences the newly formed blood vessels, but not the established ones. In agreement with our findings, VEGFA was reported to be overexpressed in several different tumor types [171, 181–183]. Anti-VEGF antibodies were implicated as potent inhibitory effectors [184, 185]. Furthermore, VEGFA expression is correlated with tumor stage and progression. It was found to be associated with high pathological grade, tumor size, lymph node metastasis, poor prognosis, resistance to chemotherapy, and poor overall survival and outcomes in several types of cancer [171, 172, 186–189].

## 5. Conclusions

The current study does confirm the association of miR-34a overexpression with RCC in our population, suggesting its potential role in pathogenesis and progression of this type of cancer. Furthermore, chromophobic RCC subtype has been postulated to attain different transcriptomics and proteomics characteristics compared to other subtypes. It has been found to have higher *MIR-34A*, *Tp53*, Ki67, and TGFB expressions. Hence, the molecular mechanism and genes involved in this particular type need to be validated in large scale multicenter study for better disease outcome and response to treatment predictions. In addition, the exact molecular interplay between the potential miR-34a target genes is still unclear and will warrant further detailed studies. One of the limitations that need to be considered is that the protein levels of the selected target genes in tumor/normal samples were not measured along with the mRNA levels. Hence, we could not suggest if the selected potential targets could be affected by translation inhibition rather than degradation in light of absence of miRNA34a-selected targets anticorrelation. Another limitation is the lack of the functional analysis either on tumor cell lines or on RCC rat models to validate the current findings and explore the detailed biological mechanisms and the potential therapeutic roles of miR-34a in RCC. This will be considered the logic next step in our ongoing research.

## Supplementary Material


**Table S1.** Experimentally validated targets of miR-34a-5p in the literature. **Table S2.** Correlation analysis of miRNA-34a and target genes and proteins in renal cell carcinoma patients. **Table S3.** Correlation analysis between expression profile and clinicopathological characteristics in renal cell carcinoma specimens. **Table S4.** Experimentally supported miR-34a-5p functional annotation. **Table S5.** Enrichment analysis of miRNA-34a targets on numerous biological process. **Table S6.** Enrichment analysis of miRNA-34a targets on cancer KEGG pathways. **Figure S1. Correlation analysis of miR-34a with the tested target genes and proteins.** Chromosomal location is shown (numbers), blue line: inverse correlation, red line: positive correlation, continuous line: *p* < 0.01, interrupted line: *p* < 0.05. **Figure S2. Schematic representation of the target genes and the predicted miR-34 binding sites.** Base pairing of miRNA-target interactions by microRNA.org web server (photo A-F) and miRTarBase v20 (photo B, G-J). **Figure S3. Protein-protein interaction using STRING network analysis.** The network is composed of 11 protein nodes and 20 edges representing protein-protein associations, with the following settings: minimum required interaction score of medium confidence (4.0), no extra interactors, and disable structure previews inside network bubbles. The network clustering coefficient was 0.82 and protein-protein interaction (PPI) enrichment *p* value was 4.77e-07. The line colors of edges indicate the type of interaction evidence; pink for experimentally determines, blue for curated database, and green for text mining.

## Figures and Tables

**Table 1:** Clinicopathological characteristics of renal cell carcinoma patients (*n* = 85).

Variables	*N*	%
Age		
20 y	5	5.9
40 y	47	55.3
60 y	33	38.8
Gender		
Females	34	40.0
Males	51	60.0
Affected side		
Right	47	44.7
Left	38	55.3
Histological type		
Clear cell RCC	47	55.3
Papillary RCC	15	17.6
Chromophobic RCC	13	15.3
Unclassified	10	11.8
Pathological grade		
Grade 1	11	12.9
Grade 2	51	60.0
Grade 3	23	27.1
Tumor size		
T1	25	29.4
T2	42	49.4
T3	18	21.2
LN involvement		
Negative	77	90.6
Positive	8	9.4
Capsular infiltration		
Negative	60	70.6
Positive	25	29.4
Vascular infiltration		
Negative	71	83.5
Positive	14	16.5
Renal pelvis infiltration		
Negative	79	92.9
Positive	6	7.1

Data are presented as *N* (number) and % (percentage); RCC: renal cell carcinoma; T: tumor size; LN: lymph node.

**Table 2:** ROC curve of miRNA-34a and target genes in renal cancer and normal tissues.

Variable(s)	Area	Standard error	*p* value	95% confidence interval	
				Lower bound	Upper bound
miR-34a	**0.854**	0.051	<0.001	0.754	0.954
MET	**0.765**	0.059	0.005	0.648	0.881
E2F3	**0.761**	0.063	0.006	0.638	0.884
SOX2	0.571	0.094	0.479	0.388	0.755
TGFB3	0.553	0.081	0.586	0.395	0.711
DFFA	0.118	0.053	0.068	0.014	0.222
TP53INP2	0.587	0.062	0.173	0.466	0.709
Combined first three markers	**0.793**	0.034	<0.001	0.727	0.859
All combined markers	**0.589**	0.030	0.014	0.530	0.648

Bold values are statistically significant at *p* < 0.05.

## Abbreviations

AUC: Area under curve
Bcl-2: B-cell lymphoma 2
CDKN1A: Cyclin dependent kinase inhibitor 1A
crRCC: Chromophobe renal cell carcinomas
ccRCC: Clear cell renal cell carcinomas
DFFA: DNA fragmentation factor subunit alpha
FFPE: Formalin-fixed paraffin embedded
HGF: Hepatocyte growth factor
ISUP: International society of urological pathology
KEGG: Kyoto encyclopedia of genes and genomes
MDM2: Mouse double minute 2 homolog
miR-34a: MicroRNA-34a
miRNAs: MicroRNAs
Ng: Nuclear grade
NSCLC: Non-small-cell lung cancer
pRCC: Papillary renal cell carcinomas
PI3K: Phosphatidylinositol 3-kinase
qPCR: Quantitative polymerase chain reaction
Cq: Quantitative cycle
ROC: Receiver operating characteristic
RCC: Renal cell carcinoma
pRB: Retinoblastoma protein
RT: Reverse transcription
SFRP1: Secreted frizzled-related protein 1
STAT: Signal transducer and activator of transcription
SOX2: Sex-determining region Y-box 2
TGFB: Transforming growth factor-beta
TP53INP: Tumor protein p53 inducible nuclear protein
UTR: Untranslated region
VEGF: Vascular endothelial growth factor
VDR: Vitamin D receptor
VMP1: Vacuole membrane protein 1.
