# Supplementary material for: MicroRNA-34a: A Key Regulator in the Hallmarks of Renal Cell Carcinoma
Source: Oxid Med Cell Longev. 2017 Sep 20;2017:3269379. doi: 10.1155/2017/3269379 (PMC5632457; doi:10.1155/2017/3269379)
Supplement: Supplementary file 2 [file 3269379.f2.docx]

| **Supplementary Table S2. Correlation analysis of miRNA-34a and target genes and proteins in renal cell carcinoma patients** | | |
| --- | --- | --- |
|  | Correlation Coefficient | *p* values |
| MET | -0.249 | 0.112 |
| E2F3 | 0.102 | 0.542 |
| SOX2 | 0.373 | 0.096 |
| TGFB3 | -0.071 | 0.716 |
| DFFA | -0.175 | 0.363 |
| TP53INP2 | -0.045 | 0.880 |
| Bcl2 | -0.134 | 0.663 |
| Tp53 | 0.165 | 0.590 |
| TGFB1 | -0.140 | 0.648 |
| Ki67 | 0.134 | 0.662 |
| VEGFA | 0.062 | 0.840 |
| Spearman's correlation analysis was used. | | |

| **Supplementary Table S3. Correlation analysis between expression profile and clinicopathological characteristics in renal cell carcinoma specimens** | | | | | | | | | | | |
| --- | --- | --- | --- | --- | --- | --- | --- | --- | --- | --- | --- |
|  | | Age | Gender | Side | HPD | Grade | T | LN | Caps | BV | Pelvis |
| miR34 | r | -0.195 | 0.071 | 0.018 | -0.106 | **-0.301** | -0.103 | 0.094 | -0.189 | -0.026 | -0.102 |
|  | P(r) | 0.185 | 0.630 | 0.901 | 0.472 | **0.037** | 0.488 | 0.527 | 0.199 | 0.863 | 0.492 |
|  | N | 85 | 85 | 85 | 85 | 85 | 85 | 85 | 85 | 85 | 85 |
|  | P* | 0.409 | 0.625 | 0.899 | **0.039** | 0.118 | 0.781 | 0.533 | 0.196 | 0.864 | 0.512 |
| *MET* | r | 0.169 | 0.024 | -0.054 | 0.126 | -0.050 | -0.016 | -0.213 | -0.026 | -0.174 | -0.062 |
|  | P(r) | 0.235 | 0.865 | 0.708 | 0.379 | 0.726 | 0.911 | 0.133 | 0.857 | 0.222 | 0.667 |
|  | N | 85 | 85 | 85 | 85 | 85 | 85 | 85 | 85 | 85 | 85 |
|  | P* | 0.488 | 0.863 | 0.704 | 0.619 | 0.122 | 0.133 | 0.135 | 0.855 | 0.223 | 0.692 |
| *E2F3* | r | 0.070 | 0.073 | -0.289 | 0.030 | -0.146 | -0.186 | -0.201 | **-0.328** | -0.123 | -0.113 |
|  | P(r) | 0.643 | 0.629 | 0.052 | 0.844 | 0.332 | 0.215 | 0.180 | **0.026** | 0.414 | 0.456 |
|  | N | 85 | 85 | 85 | 85 | 85 | 85 | 85 | 85 | 85 | 85 |
|  | P* | 0.075 | 0.624 | 0.053 | 0.148 | 0.085 | 0.255 | 0.183 | **0.028** | 0.417 | 0.565 |
| *SOX2* | r | -0.090 | 0.224 | 0.177 | 0.166 | -0.014 | 0.292 | 0.222 | 0.180 | -0.265 | **-0.413** |
|  | P(r) | 0.651 | 0.252 | 0.368 | 0.398 | 0.943 | 0.132 | 0.256 | 0.359 | 0.173 | **0.029** |
|  | N | 85 | 85 | 85 | 85 | 85 | 85 | 85 | 85 | 85 | 85 |
|  | P* | 0.502 | 0.260 | 0.378 | 0.476 | 0.664 | 0.286 | 0.28 | 0.356 | 0.192 | **0.021** |
| *TGFB3* | r | -0.074 | 0.086 | 0.050 | 0.008 | -0.041 | 0.147 | -0.158 | -0.212 | -0.300 | -0.236 |
|  | P(r) | 0.658 | 0.608 | 0.764 | 0.963 | 0.806 | 0.378 | 0.344 | 0.202 | 0.067 | 0.154 |
|  | N | 85 | 85 | 85 | 85 | 85 | 85 | 85 | 85 | 85 | 85 |
|  | P* | 0.721 | 0.616 | 0.775 | 0.818 | 0.348 | 0.272 | 0.356 | 0.207 | 70 | 0.168 |
| *DFFA* | r | -0.263 | 0.286 | -0.196 | 0.025 | -0.113 | 0.002 | -0.212 | -0.152 | **-0.419** | -0.307 |
|  | P(r) | 0.133 | 0.102 | 0.265 | 0.889 | 0.524 | 0.990 | 0.230 | 0.392 | **0.014** | 0.078 |
|  | N | 85 | 85 | 85 | 85 | 85 | 85 | 85 | 85 | 85 | 85 |
|  | P* | 0.265 | 0.102 | 0.271 | 0.842 | 0.616 | 0.999 | 0.237 | 0.401 | **0.013** | 0.079 |
| *TP53INP2* | r | 0.135 | 0.000 | 0.135 | 0.033 | -0.155 | -0.193 | 0.188 | 0.258 | 0.063 | 0.103 |
|  | P(r) | 0.571 | 1.000 | 0.571 | 0.889 | 0.514 | 0.416 | 0.428 | 0.272 | 0.793 | 0.667 |
|  | N | 85 | 85 | 85 | 85 | 85 | 85 | 85 | 85 | 85 | 85 |
|  | P* | 0.557 | 1.000 | 0.603 | 0.080 | 0.332 | 0.620 | 0.479 | 0.306 | 0.842 | 0.800 |
| Ki67 | r | -0.135 | 0.174 | 0.135 | **-0.419** | **0.690** | **0.389** | **0.351** | **0.431** | 0.285 | 0.205 |
|  | P(r) | 0.406 | 0.283 | 0.406 | **0.007** | **<0.001** | **<0.001** | **0.026** | **0.006** | 0.075 | 0.204 |
|  | N | 40 | 40 | 40 | 40 | 40 | 40 | 40 | 40 | 40 | 40 |
|  | P* | 0.343 | 0.364 | 0.343 | 0.119 | **<0.001** | **0.027** | 0.051 | **0.014** | 0.179 | 0.219 |
| Bcl2 | r | -0.115 | 0.290 | 0.000 | 0.261 | 0.266 | -0.057 | -0.192 | -0.252 | -0.190 | -0.274 |
|  | P(r) | 0.478 | 0.069 | 1.000 | 0.104 | 0.097 | 0.728 | 0.234 | 0.117 | 0.240 | 0.087 |
|  | N | 40 | 40 | 40 | 40 | 40 | 40 | 40 | 40 | 40 | 40 |
|  | P* | 0.716 | 0.14 | 1 | 0.251 | 0.161 | 0.782 | 0.256 | 0.133 | 0.338 | 0.149 |
| Tp53 | r | -0.152 | -0.269 | 0.152 | -0.229 | **-0.403** | -0.225 | -0.051 | -0.210 | -0.136 | 0.053 |
|  | P(r) | 0.350 | 0.093 | 0.350 | 0.156 | **0.010** | 0.163 | 0.757 | 0.194 | 0.401 | 0.746 |
|  | N | 40 | 40 | 40 | 40 | 40 | 40 | 40 | 40 | 40 | 40 |
|  | P* | 0.523 | 0.116 | 0.523 | **0.001** | **0.029** | 0.245 | 1 | 0.296 | 0.432 | 1 |
| TGFB1 | r | -0.123 | 0.105 | 0.032 | **0.427** | **0.441** | 0.061 | 0.061 | 0.065 | 0.123 | -0.013 |
|  | P(r) | 0.449 | 0.517 | 0.845 | **0.006** | **0.004** | 0.708 | 0.709 | 0.692 | 0.449 | 0.937 |
|  | N | 40 | 40 | 40 | 40 | 40 | 40 | 40 | 40 | 40 | 40 |
|  | P* | 0.354 | 0.145 | 0.752 | **0.035** | **0.034** | 0.399 | 0.263 | 0.949 | 0.744 | 0.934 |
| VEGFA | r | -0.144 | 0.222 | -0.005 | 0.241 | **0.563** | 0.160 | 0.096 | 0.037 | -0.032 | -0.301 |
|  | P(r) | 0.374 | 0.168 | 0.976 | 0.135 | **<0.001** | 0.325 | 0.555 | 0.822 | 0.846 | 0.059 |
|  | N | 40 | 40 | 40 | 40 | 40 | 40 | 40 | 40 | 40 | 40 |
|  | P* | 0.256 | 0.181 | 0.443 | 0.123 | **0.001** | 0.631 | 0.545 | 0.537 | 0.747 | 0.099 |
| r; spearman's correlation coefficient. P(r); p value of correlation coefficient. N; number of specimens. P*; Mann-Whitney U and Kruskal-Wallis tests were used for comparing gene expression between groups, while Chi-square (χ^2^) test was used for protein expression. Statistical significance at *p* < 0.05. Side; affected renal side, HPD; histopathological diagnosis, T; tumor size, LN; lymph node involvement, Caps; capsular infiltration, BV; vascular infiltration, Pelvis; pelvic infiltration. | | | | | | | | | | | |
