# Supplementary material for: MicroRNA-34a: A Key Regulator in the Hallmarks of Renal Cell Carcinoma
Source: Oxid Med Cell Longev. 2017 Sep 20;2017:3269379. doi: 10.1155/2017/3269379 (PMC5632457; doi:10.1155/2017/3269379)
Supplement: Supplementary file 6 [file 3269379.f6.docx]

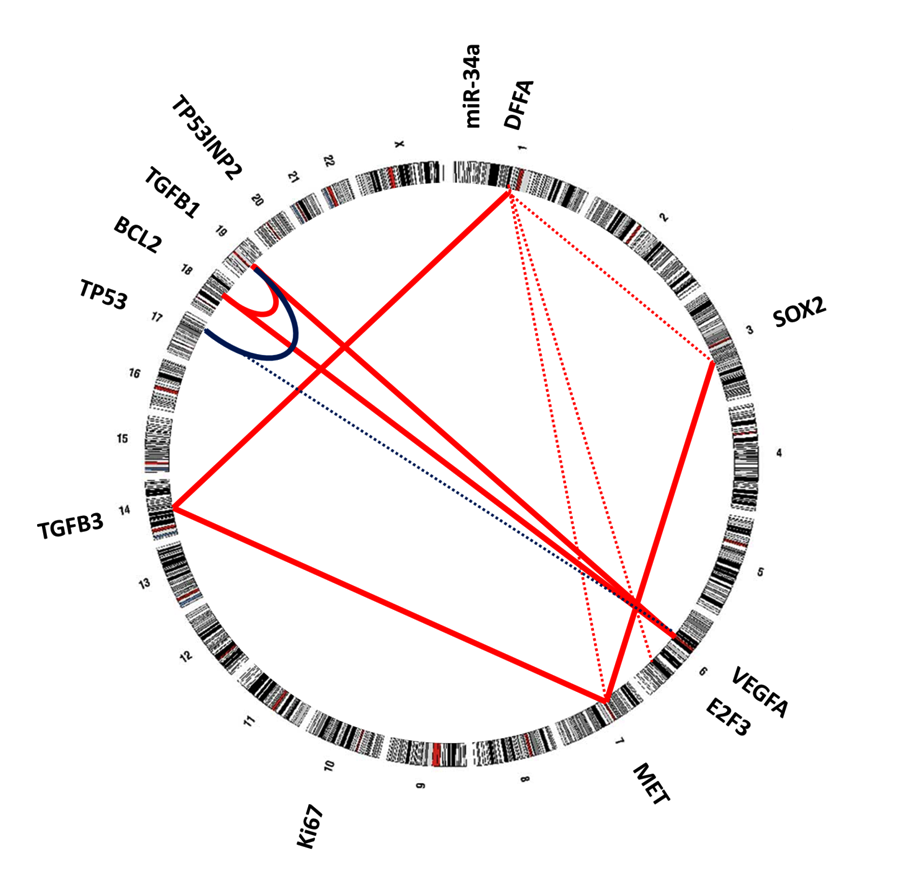


**Figure S1. Correlation analysis of miR-34a with the tested target genes and proteins.** Chromosomal location is shown (numbers), blue line: inverse correlation, red line: positive correlation, continuous line: *p* < 0.01, interrupted line: *p* < 0.05.
