# Supplementary material for: MicroRNA-34a: A Key Regulator in the Hallmarks of Renal Cell Carcinoma
Source: Oxid Med Cell Longev. 2017 Sep 20;2017:3269379. doi: 10.1155/2017/3269379 (PMC5632457; doi:10.1155/2017/3269379)
Supplement: Supplementary file 7 [file 3269379.f7.docx]

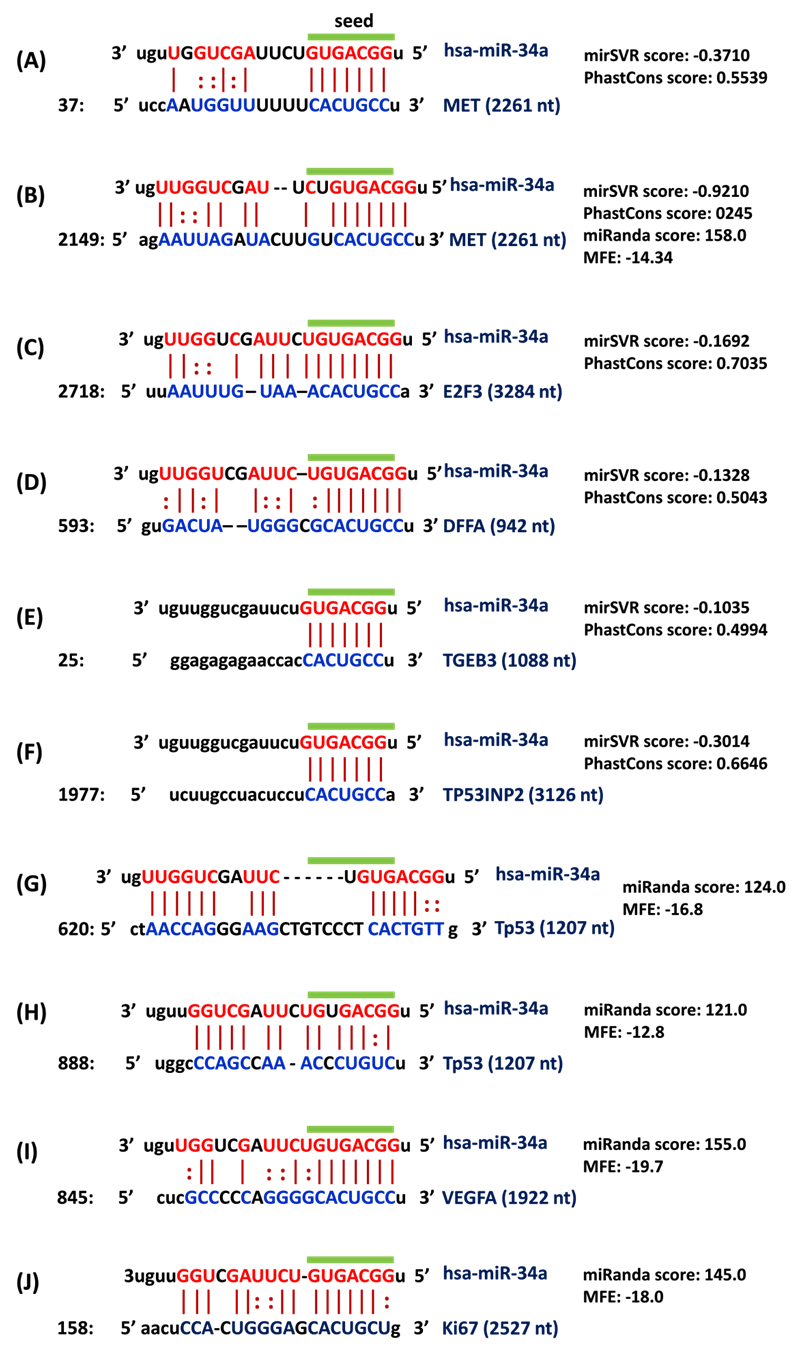


**Figure S2. Schematic representation of the target genes and the predicted miR-34 binding sites.** Base pairing of miRNA-target interactions by microRNA.org web server (photo A-F) and miRTarBase v20 (photo B, G-J).
