# Supplementary material for: MicroRNA-34a: A Key Regulator in the Hallmarks of Renal Cell Carcinoma
Source: Oxid Med Cell Longev. 2017 Sep 20;2017:3269379. doi: 10.1155/2017/3269379 (PMC5632457; doi:10.1155/2017/3269379)
Supplement: Supplementary file 8 [file 3269379.f8.docx]

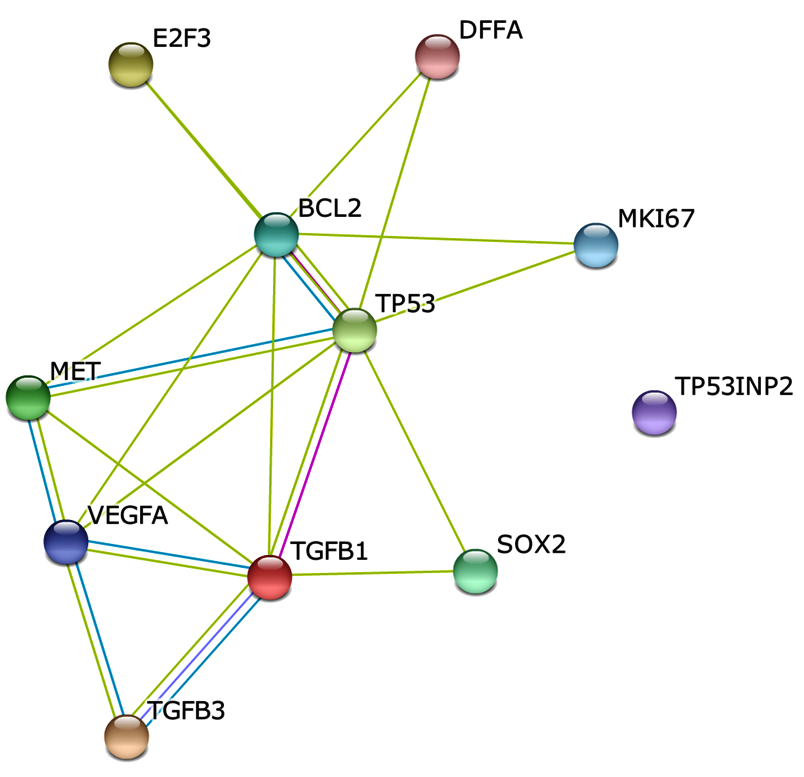


**Figure S3. Protein-protein interaction using STRING network analysis.**

The network is composed of 11 protein nodes and 20 edges representing protein-protein associations, with the following settings: minimum required interaction score of medium confidence (4.0), no extra interactors, and disable structure previews inside network bubbles. The network clustering coefficient was 0.82 and protein-protein interaction (PPI) enrichment *p* value was 4.77e-07. The line colors of edges indicate the type of interaction evidence; pink for experimentally determines, blue for curated database, and green for text mining.
